# A predictive scoring instrument for tuberculosis lost to follow-up outcome

**DOI:** 10.1186/1465-9921-13-75

**Published:** 2012-09-02

**Authors:** Teresa Rodrigo, Joan A Caylà, Martí Casals, José Mª García-García, José A Caminero, Juan Ruiz-Manzano, Rafael Blanquer, Rafael Vidal, Neus Altet, José L Calpe, Antón Penas

**Affiliations:** 1 Programa Integrado de Investigación en Tuberculosis (PII TB) de la Sociedad Española de Neumología y Cirugía Torácica (SEPAR), Barcelona, Spain; 2 Unidad de Investigación de Tuberculosis de Barcelona, Servicio de Epidemiología de la Agencia de Salud Pública de Barcelona, Barcelona, Spain; 3 Fundación Respira de la SEPAR, Barcelona, Spain; 4 Hospital San Agustín, Avilés, Asturias, Spain; 5 Hospital General Universitario de Gran Canaria Dr. Negrín, Canary Islands, Spain; 6 Hospital Universitario Germans Trías y Pujol de Badalona, Badalona, Spain; 7 Hospital Universitario Dr. Peset de Valencia, Valencia, Spain; 8 Hospital Vall D'Hebrón de Barcelona, Barcelona, Spain; 9 Unidad de Prevención y Control de Tuberculosis de Barcelona, Barcelona, Spain; 10 Hospital Marina Baixa de Alicante, Alicante, Spain; 11 Complejo Hospitalario Xeral-Calde, Lugo, Spain; 12 Centro de Investigación Biomédica en Red de Epidemiología y Salud Pública (CIBERESP), Barcelona, Spain; 13 CIBER de Enfermedades Respiratorias (CIBERES), Barcelona, Spain; 14 International Union Against Tuberculosis and Lung Disease, París, France; 15 Departament de Salut Pública, Universitat de Barcelona, Barcelona, Spain

**Keywords:** Lost to follow-up outcome, Scoring system, Tuberculosis, Adherence, Predictors

## Abstract

**Background:**

Adherence to tuberculosis (TB) treatment is troublesome, due to long therapy duration, quick therapeutic response which allows the patient to disregard about the rest of their treatment and the lack of motivation on behalf of the patient for improved. The objective of this study was to develop and validate a scoring system to predict the probability of lost to follow-up outcome in TB patients as a way to identify patients suitable for directly observed treatments **(**DOT) and other interventions to improve adherence.

**Methods:**

Two prospective cohorts, were used to develop and validate a logistic regression model. A scoring system was constructed, based on the coefficients of factors associated with a lost to follow-up outcome. The probability of lost to follow-up outcome associated with each score was calculated. Predictions in both cohorts were tested using receiver operating characteristic curves (ROC).

**Results:**

The best model to predict lost to follow-up outcome included the following characteristics: immigration (1 point value), living alone (1 point) or in an institution (2 points), previous anti-TB treatment (2 points), poor patient understanding (2 points), intravenous drugs use (IDU) (4 points) or unknown IDU status (1 point). Scores of 0, 1, 2, 3, 4 and 5 points were associated with a lost to follow-up probability of 2,2% 5,4% 9,9%, 16,4%, 15%, and 28%, respectively. The ROC curve for the validation group demonstrated a good fit (AUC: 0,67 [95% CI; 0,65-0,70]).

**Conclusion:**

This model has a good capacity to predict a lost to follow-up outcome. Its use could help TB Programs to determine which patients are good candidates for DOT and other strategies to improve TB treatment adherence.

## Background

The highest priority for control of tuberculosis (TB) is to achieve an early diagnosis and satisfactory treatment adherence [1].

To ensure satisfactory adherence, since the 1990s [2] the World Health Organization (WHO) has recommended the use of directly observed treatment (DOT). Likewise one of components of the Stop TB Strategy is pursuing high-quality DOT expansion and enhancement as well as special attention to certain population groups and special situations that are associated with a higher TB risk. The first step in addressing the needs of risk groups is recognition and acknowledgement of their existence and their special requirements [3]. Nonetheless it exists still inconvenience in the implementation of DOT, since it can be difficult and costly [4] despite evidence of cost-effectiveness [5].

A valuable strategy to ensure treatment compliance is to determine the patient characteristics associated with satisfactory adherence and to identify patients who are at risk of lost to follow-up outcome. Other methods of control, such as early detection of patients at high risk, also contribute to reduce this risk [6].

The objective of this study was to describe and validate a predictive score which can be used by healthcare professionals to quantify the risk of lost to follow-up outcome of a patient at the time of diagnosis, as a way to identify patients suitable for DOT and other interventions to improve treatment adherence.

## Methods

### Study setting

A multi-centered, national and population-based prospective study, comprised of derivation and validation cohorts, was performed involving 43 collaborating professionals at 35 healthcare centers in Spain from January 1, 2006 to December 31, 2009.

### Case identification and follow-up

The study included all patients, diagnosed with TB over 18 years of age with smear-positive TB, or smear-negative sputum with a culture positive for *Mycobacterium tuberculosis*, extrapulmonary TB disease with caseating granuloma identification by histology, and patients with clinical, radiological, epidemiological or laboratory suspicion of TB, for whom the clinician prescribed anti-TB treatment and was followed until the end of treatment. A verbal consent form, approved by our Research Ethics Committee (see below), was dictated to the patient and signed by the treating physician.

Exclusion criteria included patients with known drug resistance and those with a contraindication to start standard treatment of three or four drugs.

The cases follow-up was according to an evaluation schedule (Table 1).

**Table 1:** Evaluation schedule for tuberculosis patients included in the PII TB cohort (2006–2009)

	**1st visit**	**2nd visit**	**3rd visit**	4th visit*
	**Diagnosis**	**Month 2**	**Month 6**	**Month 9, 12 or 18 (optional)**
**Inclusion/exclusion criteria**	X			
**Sociodemographic information**	X			
**Smoking and use of alcohol**	X			
**Anthropometrics data**	X	X	X	X
**Clinical history**	X			
**Type of diagnostic test**	X			
**Pharmalogical treatment**	X	X	X^*^	X
**Clinical evolution**		X	X	X
**Treatment adherence**		X	X	X
**Sputum sample**	X	X	X	X
**Drug sensitivity testing**		X		
**Treatment result**			X	X

*If treatment continued past 6 months.

Patient information was prospective and stored in an electronic case report form with a standardized questionnaire generated for each patient in the entire cohort and available online, with a username and password specified for each collaborating study investigator. The program was tested for three months and some changes and eliminations were made before the study. The data manager was responsible for reviewing and completing anomalous or missing data. Investigators and clinicians communicated about case report completion and the database by means of telephone and email.

The database contained information regarding sociodemographics, smoking habits and alcohol use, anthropometric information, clinical history, diagnostic methods, drug sensitivity testing, pharmalogical treatment, clinical evolution, and treatment adherence and outcome. Living situation was considered “alone” when the patient had housing and “institutionalized” when the patient lived in a closed institution, such as a residence hall, prison, mental hospital, etc. Country of origin was classified as native or immigrant. We did not classify immigrants into sub-groups because of the great diversity in country of origin and the few number of cases from each country. The variable "understanding" was systematically reviewed for each patient by a physician based on the presence of linguistic or cultural barriers.

*Lost to follow-up outcome* (defaulter in the past) was defined according to European recommendations [7,8] as interruption of treatment for any reason for more than two months, non-completion of treatment within 9 months when the patient is placed on a six-month regimen, or drug intake of < 80% of the prescribed dose.

The derivation cohort included patients diagnosed with TB between January 1, 2006 and February 28, 2007 and compared subjects who completed treatment to lost to follow-up subjects (those who died or moved during treatment, as well as those who did not respond to treatment were not included). The validation cohort was comprised of patients diagnosed with TB between March 1, 2007 and December 31, 2009, based on similar simple size to the derivation cohort; no patients were excluded from the validation group because their outcome was unknown at the time of inclusion.

According to the International Directives for Ethical Review of Epidemiological Studies (Council for the International Organizations of Medical Sciences, Geneva 1991) and to recommendations about ethical aspects of epidemiological research by the Spanish Epidemiology Society, this study about predicting factors of bad adherence to TB treatment (derivation cohort) was approved by the Research Ethics Committee of Teknon Medical Center, Barcelona. All information that identified the study subjects was confidential and was managed according to Law 15/1999 Protection of Data about Personal Character.

The investigators do not have conflicts of interest.

### Data analysis

A bivariate analysis was used to identify the risk factors associated with lost to follow-up outcome for the derivation cohort, comparing subjects who were cured plus those who completed treatment to lost to follow-up subjects. All variables without presence of colinearity were included in the logistic regression model using the stepwise method to determine predictors of lost to follow-up outcome (p ≤ 0·05). *Odds ratio* (OR) with a 95% confidence intervals (CI) was calculated as a measurement of association. Patients with the lowest rates of lost to follow-up outcome were considered the reference category. P-values equal to or below 0·05 were considered statistically significant.

Independent predicting factors were assigned a point value by dividing its coefficient from the logistic regression by a lowest coefficient and rounded to the nearest whole number. Each patient was assigned a total number of points and a predictor value using the model for anti-TB lost to follow-up outcome. Sensitivity, specificity, positive predictive value (PPV) and negative predictive value (NPV) were calculated for each possible value.

Reliability (concordance between predicted and observed results) was analyzed in the following manner:

By comparing coefficients from the regression model, which adjusted the validation group to the original derivation model using a z-score test.

By grouping patients by terciles of predicted risk and comparing each tercil of predicted of lost to follow-up outcome with observed lost to follow-up outcome. Goodness of fit was assessed using the Hosmer-Lemeshow test.

The model’s discrimination was analyzed by using receiver operating characteristics (ROC) curves [9] to compare the distribution of predictors for subjects who did and did not lost to follow-up outcome. The curves were constructed from the point intersection of the derivation and validation groups. Area under the curve (AUC) with 95% CI was calculated for both ROC curves to determine if the predicting model was better than one due to chance.

Analyses were performed using SPSS statistical package, version 18.0 (SPSS Inc, Chicago, IL USA) and R statistical package, version 2.8.1 (The R Foundation for Statistical Computing, Vienna, Austria). The χ^2^ test was used to compare qualitative variables and Fisher’s exact test was used when expected values were less than 5. EpiR [10] was used to calculate diagnostic tests.

## Results

A total of 3079 subjects were included in the study: 1490 subjects in the derivation cohort and 1589 subjects in the validation group. Table 2 provides a summary of the characteristics and risk factors associated with poor adherence of both groups. The lost to follow-up outcome was similar between the derivation and validation cohorts (6.4% vs 6.5%; p = 0.974).

**Table 2:** Comparison of the clinical and epidemiological characteristics of the derivation and validation cohorts

**Variables**	**Total**	**Derivation**^**a**^	**Validation**^**a**^	**p-value**^**b**^
		**N (%)**	**N (%)**	
**Country:**				0.002
Spain	2080 (67.6%)	1048 (70.3%)	1032 (65.1%)	
Other	995 (32.4%)	442 (29.7%)	553 (34.9%)	
**Understanding:**				0.003
Good	2566 (91.7%)	1266 (90.1%)	1300 (93.3%)	
Poor	232 (8.29%)	139 (9.89%)	93 (6.68%)	
**Living situation:**				0.250
Alone	312 (10.5%)	157 (10.7%)	155 (10.3%)	
Homeless or institutionalized	92 (3.10%)	54 (3.69%)	38 (2.52%)	
Group	376 (12.7%)	189 (12.9%)	187 (12.4%)	
Family	2191 (73.7%)	1062 (72.6%)	1129 (74.8%)	
**Intravenous drug use:**				<0.001
No	1399 (45.5%)	872 (58.5%)	527 (33.2%)	
Yes	49 (1.59%)	21 (1.41%)	28 (1.77%)	
Unknown	1627 (52.9%)	597 (40.1%)	1030 (65.0%)	
**Age (years):**				0.449
18-30	986 (32.3%)	497 (33.4%)	489 (31.3%)	
31-50	1233 (40.4%)	596 (40.0%)	637 (40.8%)	
>50	834 (27.3%)	397 (26.6%)	437 (28.0%)	
**Human immunodeficiency virus infection:**				<0.001
No	2284 (74.3%)	1060 (71.1%)	1224 (77.2%)	
Yes	148 (4.81%)	66 (4.43%)	82 (5.17%)	
Unknown	643 (20.9%)	364 (24.4%)	279 (17.6%)	
**Use of directly observed treatment:**		0.017		
No	2626 (91.1%)	1338 (89.8%)	1288 (92.4%)	
Yes	258 (8.95%)	152 (10.2%)	106 (7.60%)	
**Sex:**				0.476
Male	1945 (64.0%)	920 (63.4%)	1025 (64.7%)	
Female	1092 (36.0%)	532 (36.6%)	560 (35.3%)	
**Country- Healthcare**				<0.001
Primary care	511 (17.1%)	268 (18.0%)	243 (16.1%)	
Urgent care	1461 (48.8%)	682 (45.8%)	779 (51.7%)	
Specialist	476 (15.9%)	210 (14.1%)	266 (17.7%)	
Unknown'	548 (18.3%)	330 (22.1%)	218 (14.5%)	
**Employment:**				<0.001
Disabled	98 (3.39%)	68 (4.70%)	30 (2.08%)	
Employed	1781 (61.6%)	901 (62.3%)	880 (60.9%)	
Unemployed	547 (18.9%)	257 (17.8%)	290 (20.1%)	
Retired	464 (16.1%)	220 (15.2%)	244 (16.9%)	
**Previous anti-tuberculosis treatment:**				0.304
No	2728 (91.5%)	1320 (91.0%)	1408 (92.1%)	
Yes	252 (8.46%)	131 (9.03%)	121 (7.91%)	
**Lost to follow-up outcome**				0.974
No	2814 (93.5%)	1332 (93.5%)	1482 (93.5%)	
Yes	195 (6.48%)	92 (6.46%)	103 (6.50%)	

^a^Data from the derivation group was used to create the model and data from the validation group was used to valídate the model.

^b^p values according to χ^2^ test with correction for continuity.

### Derivation model cohort

The following independent predictors of lost to follow-up outcome were identified from the multivariate analysis: country of residence, living alone, living in an institution, previous anti-TB treatment, poor patient understanding, intravenous drug use (IDU) and unknown IDU status. Using the logistic regression coefficients, 4 points were assigned to IDU subjects, 2 points were assigned to those living in an institution, those who had previously received anti-TB treatment and with poor understanding, and one point was assigned to immigrants, patients who lived alone and those with an unknown IDU status. A score of 11 points represented the worst prognosis for treatment compliance (Table 3).

**Table 3:** Prognostic score for lost to follow-up outcome according to a multivariate analysis of the derivation cohort

**Variables**		**β**	**Se (β)**	**p-value**	**OR**^**a**^	**95% CI**^**b**^	**Score**^**c**^
**Country of origin**	**Immigrant**	0.71	0.330	0.031	2.03	(1.06 - 3.88)	**1**
**Living situation**	**Alone**	0.85	0.410	0.037	2.35	(1.05 - 5.26)	**1**
	**Institution**	1.56	0.515	0.002	4.79	(1.74 - 13.14)	**2**
**Previous treatment**	**Yes**	1.03	0.395	0.009	2.80	(1.29 - 6.08)	**2**
**Understanding**	**Poor**	1.07	0.363	0.003	2.93	(1.44 - 5.98)	**2**
**Intravenous drug use**	**Yes**	2.25	0.642	<0.001	9.51	(2.70 - 33.47)	**4**
	**Unknown**	0.64	0.306	0.034	1.90	(1.04 - 3.47)	**1**

^a^OR: *odds ratio.*

^b^CI: Confidence interval.

β: Regression coefficient.

Se: Standard error.

^c^Regression coefficient divided by the lowest value coefficient (0.64), rounded to the nearest whole number; used to determine the point value assigned to each independent predictor.

Sensitivity, specificity, PPV and NPV results can be found in Table 4. Note the positive relationship between PPV and the point score.

**Table 4:** Score evaluation for derivation and validation groups

	**Derivation group**^**a**^				**Validation group**^**a**^			
**Predictive score**	**Sensitivity**	**Specificity**	**PPV**^**b**^	**NPV**^**c**^	**Sensitivity**	**Specitifity**	**PPV**	**NPV**
**≥ 0**	100.00	0.00	6.5	….	100.00	0.00	6.5	….
**≥ 1**	83.70	54.65	11.3	98.0	93.20	16.69	7.2	97.3
**≥ 2**	46.74	80.71	14.3	95.6	65.05	67.36	12,1	96.5
**≥ 3**	34.78	90.54	20.3	95.3	26.21	89.84	15.2	94.6
**≥ 4**	13.04	97.30	25.0	94.2	11.65	95.76	16.0	94.0
**≥ 5**	7.61	98.80	30.4	93.9	4.85	98.99	25.0	93.8

^a^The derivation group included 1490 patients and the validation group included 1589 patients.

^b^PPV: positive predictive value (probability of lost to follow-up outcome).

^c^NPV: negative predictive value (probability of no lost to follow-up outcome).

### Validation cohort

A multivariate analysis was performed to estimate the coefficients of the above predictors of lost to follow-up outcome. No statistically significant differences existed between coefficients of each cohort, except for unknown IDU status (Table 2).

Similar results were observed for sensitivity, specificity, PPV and NPV tests (Table 4).

We were unable to use deciles of risk for the Hosmer-Lemeshow test of the derivation cohort because some expected values were under 5. Therefore, observed and expected values were grouped into 3 categories, which were found to be similar (p = 0.209).

**Figure 1:**
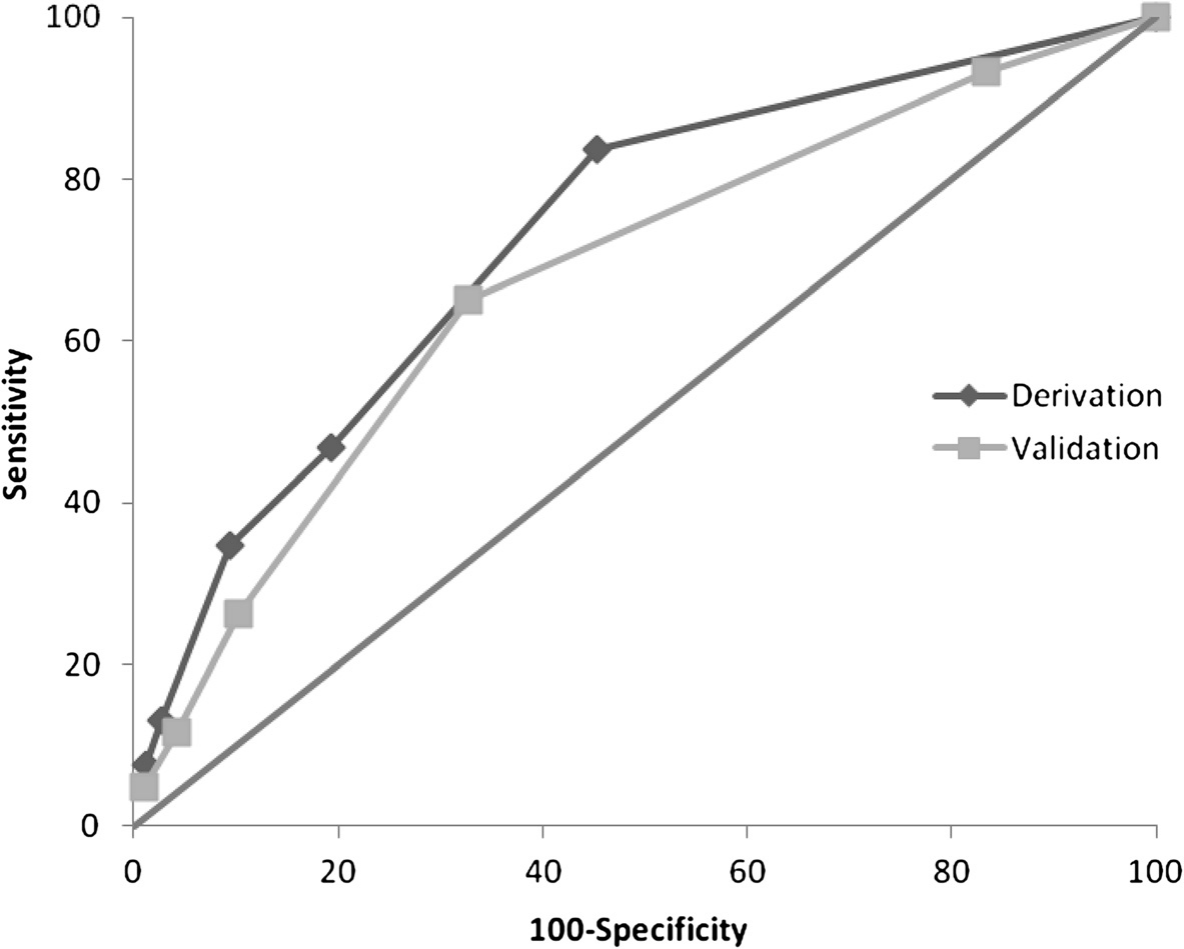
Receiver operating characteristics curves: comparison between derivation and validation cohorts.

Area under the ROC curve for the derivation and validation groups were 0.73 (95% IC of 0.70-0.75) and 0.67 (95% IC of 0.65-0.70), respectively (Figure 1). No statistically significant differences were noted.

The probability of lost to follow-up outcome by observed score can be found in Table 5. Increasing risk of lost to follow-up outcome corresponds to an increased score (p < 0.001).

**Table 5:** Probability of lost to follow-up outcome for tuberculosis patients in the PII TB cohort according to their predictive score (2006–2009)

**Predictive score**	**Lost to follow-up outcome / Total**	**Rate of lost to follow-up outcome (%)**	**OR (95% CI)**^**a**^	**p value**
0^b^	22 / 998	2.2	1	….
1	63 / 1163	5.4	2.54 (1.55 - 4.15)	<0.0001
2	51 / 516	9.9	4.86 (2.91 - 8.11)	<0.0001
3	35 / 213	16.4	8.72 (4.99 - 15.22)	<0.0001
4	12 / 80	15	7.82 (3.71 - 16.49)	<0.0001
5	12 / 43	28	17.17 (7.80 - 37.80)	<0.0001

^a^OR: *odds ratio*, *CI*: confidence interval ^b^ Reference category: indicates unable to calculate.

As seen in Table 6, a score of 2 or more points can be considered criteria for DOT implementation, which represents 28.2% of our study population or 56.4% of the subjects with a lost to follow-up outcome. A score of 3 or more points represents 11.1% of our study population and 30.2% of those with lost to follow-up outcome.

**Table 6:** Percentage of total patients undergoing directly observed therapy and proportion of lost to follow-up patients according to their predictive score

**Predictive score**	**% patients undergoing DOT**	**% lost to follow-up patients in DOT**
**≥ 0**	100	100
**≥ 1**	66.8	88.7
**≥ 2**	28.2	56.4
**≥ 3**	11.1	30.2
**≥ 4**	4.0	12.3
**≥ 5**	1.4	6.1

*DOT*: Directly Observed Therapy.

## Discussion

As our study results show, we were able to obtain and validate a predictive score for TB treatment adherence for a large cohort of new TB cases undergoing standard TB treatment. The score could be easy for a clinician to calculate situation risk of lost to follow-up at the time of diagnosis because the evaluation of it is dependent on only clinical and epidemiological characteristics, not on results of complimentary tests. The results of the statistical model indicate this predicting system is valid to evaluate the probability of lost to follow-up outcome before treatment is even initiated, as shown by the positive relationship between rate of lost to follow-up and point score.

Other components of the Stop TB Strategy are contributing to health system strengthening and engaging all care providers [3] but the implementation of DOT is not possible in many countries and is not the rule in developed countries. This TB score is a useful to determine the proportion of patients for who DOT should be considered, depending of available resources of the TB program.

The independent predictors factors of lost to follow-up outcome such as living situation or previous TB treatment were also consistent to those of other studies [11-13]. Other predictive models have been created in recent years have to estimate the risk of certain diseases [14-17], including some for TB (on quality of respiratory health [18], preventative isolation [19], severity pulmonary TB by means of chest x-ray [20], clinical course [21] and risk of multi-drug resistance [22]), but to our knowledge none have been created for the risk of lost to follow-up outcome.

Our score takes into account some characteristics, such as country of origin. Some studies have shown that the immigrant patient population does not meet the treatment adherence objectives described by the WHO, but the native patient population does [23]. Furthermore, the percentage of annual TB cases among immigrant populations of various countries is higher [24-27]. Thus it is important a predictive score that provides the probability of lost to follow-up outcome at the time of diagnosis considers characteristics such as country of residence, living alone or in an institution, history of TB or IDU, or poor understanding. It should be noted that poor patient understanding refers to not only a language barrier, but also difficulty in understanding treatment instructions which can occur in native and immigrant population [24].

According to our model, the probability of lost to follow-up outcome corresponds with a significantly elevated risk of all point categories and also increases with the total points acquired by each patient. Our analyses that examined concordance between the predicted and observed values yielded similar results and the CI of the ROC curves did not detect any significant differences. This confirms the reliability and discrimination capacity of our model.

Treatment adherence is directly related to many factors, such as gender, poverty, economic difficulty, social context, healthcare services, personal interpretation of the disease [28], drug addiction, country of origin [29], history of TB [23], alcoholism, homelessness and HIV infection [30]. The score of our model allows a clinician to determine which TB patients have a higher risk of lost to follow-up by a simple score ≥2. The clinician can then decide what measure should be taken to improve treatment adherence [21], such as scheduling additional clinical visits, providing more information about TB, providing family or social support, reducing drug prescription costs, hasten future clinical visits, improving communication between involved healthcare professionals, collaborating with public health personnel or community health workers [31] and finally accurately allocating DOT resources to the patients who need it the most.

Systematic DOT is a proven effective intervention to achieve treatment adherence [32]. For example, treatment compliance rates have reached over 95% in subgroups for which DOT was made a priority [33]. As consequence of that DOT is more costly than self-administered treatment [5], all TB control programs must use efficiently their resources.

The score estimated in our study reflects the study setting, with a high quality and uniform healthcare system throughout the country. The score calculated in other settings may vary according to different patient characteristics and healthcare systems. In the setting of limited healthcare distribution, the score can represent the side of the medal dedicated to healthcare and can indicate where improvements should be made. Current studies such as this should be reviewed by control programs to facilitate the use of predicting factors of treatment non-compliance to identify high risk subgroups. A predictive score can be extremely helpful to best direct DOT interventions.

One study limitation is that patients with known drug resistance and those with a contraindication to start standard treatment of three or four drugs were excluded because the first study cohort (derivation cohort) was also used to study treatment completion among TB patients [23]. Patients with known drug resistance were excluded because they required longer treatment, had poor compliance and represented few cases, which could have distorted the results.

Consequently, the second cohort (validation cohort) also followed the same exclusion criteria. Other limitation is the use of terciles of risk for the Hosmer-Lemeshow test instead of deciles of risk because of an insufficiently large sample size. Nonetheless, we still consider the model valid because the observed and expected values were similar.

## Conclusion

We have described a predictive model to classify and grade the risk of anti-TB lost to follow-up outcome. Given adequate funding, our model will aid in the identification of patients in need of DOT and other interventions to improve treatment adherence.

## Abbreviations

AUC: Area under the curve; CI: Confidence intervals; CIBERESP: Centro de Investigación Biomédica en Red de Epidemiología y Salud Pública; DOT: Directly observed treatment; IDU: Intravenous drugs use; NPV: Negative predictive value; OR: *Odds ratio*; PII: TB - Programa Integrado de Investigación en Tuberculosis; PPV: Positive predictive value; ROC: Receiver operating characteristics; SEPAR: Sociedad Española de Neumología y Cirugía Torácica; TB: Tuberculosis; WHO: World Health Organization.

## Competing interests

The authors declare that they have no financial or no-financial competing interests.

## Authors' contributions

TR and JC conceived of the study, and participated in its design and coordination, and helped to draft the manuscript. Also carried out the literature search. MC and JC performed the statistical analysis. The principal authors (TR, JC, MC, JMG, JAC, JR, RB, RV, NA, JLC and AP) have taken part in the data collection, data interpretation and writing. The Working Group on Completion of Tuberculosis Treatment in Spain have taken part in the collection of the cases and they have reviewed the paper. All authors have read and approved the final manuscript.

## Working group on completion of tuberculosis treatment in Spain

L Anibarro, MD (Unidad de Tuberculosis de Pontevedra, Pontevedra); F Álvarez (Hospital San Agustín, Avilés); M Barrón, MD (H San Millán-San Pedro, Logroño); A. Bustamante, PhD (H Sierrallana, Torrelavega); F Cañas, MD (H Insular de Gran Canaria); E Cases, PhD (H Universitario La Fe, Valencia); ML De Souza, MD (Unidad Prevención y Control Tuberculosis, Barcelona); J Gallardo (H General de Guadalajara, Guadalajara); M Gallego, MD (Corporación Sanitaria Parc Taulí, Sabadell); FJ García, MD (H Universitario de la Princesa, Madrid); JA Gullón, PhD (Hospital San Agustín, Avilés); MA Jiménez, MD (Unidad Prevención y Control Tuberculosis, Barcelona); T Lloret, PhD (H General Universitario de Valencia, Valencia); M Marín, PhD (H General de Castellón, Castellón); A Martínez, PhD (H Marina Baixa de Alicante, Alicante) JF Medina, PhD (H Universitario Virgen del Rocío, Sevilla); C Melero, PhD (H 12 de Octubre, Madrid); C Milà, MD (Unidad Prevención y Control Tuberculosis, Barcelona); I Mir, MD (H Son Llatzer, Palma de Mallorca); V Moreno, MD (H Carlos III, Madrid); C Muñoz (H Clínico Universitario de Valencia, Valencia); T Pascual, MD (H de Cabueñes, Gijón); P Sánchez, MD (H del Mar, Barcelona); D. Sande, MD (Unidad de Tuberculosis de Pontevedra, Pontevedra); E Valencia, PhD (H Carlos III, Madrid); A Vargas, PhD (H Universitario Puerto Real, Cádiz).
